# PHOTOGRAMMETRY: A PROPOSAL OF OBJECTIVE ASSESSMENT OF CHEST WALL IN ADOLESCENT IDIOPATHIC SCOLIOSIS

**DOI:** 10.1590/1984-0462/;2019;37;2;00001

**Published:** 2019-07-22

**Authors:** Anderson Sales Alexandre, Evandro Fornias Sperandio, Liu Chiao Yi, Josy Davidson, Patrícia Rios Poletto, Alberto Ofenhejm Gotfryd, Milena Carlos Vidotto

**Affiliations:** a Universidade Federal de São Paulo, Santos, SP, Brasil.; b Hospital Israelita Albert Einstein, São Paulo, SP, Brasil.

**Keywords:** Scoliosis, Adolescent, Thoracic wall, Spine, Photogrammetry, Escoliose, Adolescente, Parede torácica, Coluna vertebral, Fotogrametria

## Abstract

**Objective:**

To evaluate the chest wall shape in patients with adolescent idiopathic scoliosis (AIS) in comparison to healthy subjects and the association between the chest wall shape with the spine deformity and lung function in patients with AIS.

**Methods:**

This cross-sectional study enrolled 30 AIS patients and 20 healthy subjects aged 11-18 years old. The Cobb angle evaluation was performed in AIS patients. The chest wall shape was assessed by the photogrammetry method, using the Postural Assessment Software (PAS). We created thoracic markers shaped as angles (A) and distances (D), as follows: A2 (right acromion/xiphoid/left acromion), A4L (angle formed between the outer point of the smallest waist circumference and its upper and lower edges on the left side), A7 (angle formed by the intersection of the tangent segments of the upper and lower scapulae angles), D1R/D1L [distance between the xiphoid process and the last false rib on the right (R) and left (L) sides], and D3 (distance between xiphoid process and anterior superior iliac spine).

**Results:**

The thoracic markers A2 and A7 were significantly higher, while the A4L and D1R/D1L were significantly reduced in the AIS group compared to the control. Moderate correlations were found between: A2 and the main and proximal thoracic Cobb angles (r=0.50, r=0.47, respectively); D1R/D1L and the main thoracic Cobb angle (r=- 0.40); and the forced expiratory volume in the first second (FEV_1_) and D3R (r=0.47).

**Conclusions:**

The photogrammetry method was able to detect chest wall changes in AIS patients, besides presenting correlation between Cobb angles and lung function.

## RESUMO

**Objetivo:**

Avaliar o formato da caixa torácica em pacientes com escoliose idiopática do adolescente (EIA), comparando-os com indivíduos saudáveis e analisar a associação do formato da caixa torácica com a deformidade da coluna vertebral e função pulmonar em pacientes com EIA.

**Métodos:**

Estudo transversal que avaliou 30 pacientes com EIA e 20 indivíduos saudáveis com idade entre 11 e 18 anos. O ângulo de Cobb foi avaliado em pacientes com EIA. O formato da caixa torácica foi analisado pelo método da fotogrametria, utilizando o Software para Avaliação Postural (SAPO). Foram criados marcadores torácicos descritos como ângulos (A)e distâncias (D): A2 (acrômio direito/processo xifoide/acrômio esquerdo), A4E (ângulo formado entre o ponto externo da menor circunferência da cintura e suas bordas superior e inferior do lado esquerdo), A7 (ângulo formado pela interseção das retas tangentes aos ângulos superior e inferior das escápulas), D1D/D1E [distância entre o processo xifoide e a última costela falsa nos lados direito (D) e esquerdo (E)] e D3 (distância entre o processo xifoide e a espinha ilíaca anterossuperior).

**Resultados:**

Os marcadores torácicos A2 e A7 foram significativamente maiores, enquanto o A4E e o D1D/D1E foram significativamente menores no grupo EIA em relação ao controle. Foram encontradas correlações moderadas entre: A2 e os ângulos de Cobb torácico principal e proximal (r=0,50 e r=0,47, respectivamente); D1D/D1E e o ângulo de Cobb torácico principal (r=-0,40); e o volume expiratório forçado no primeiro segundo (VEF_1_) e D3D (r=0,47).

**Conclusões:**

O método da fotogrametria detectou alterações na caixa torácica de pacientes com EIA, além de apresentar correlações significativas entre os ângulos de Cobb e a função pulmonar.

## INTRODUCTION

Adolescent idiopathic scoliosis (AIS) is a deformity characterized by lateral deviation in the frontal plane, thoracic hypokyphosis in the sagittal plane and rotation in the transverse plane.^1^ Idiopathic scoliosis represents about 90% of all scoliosis cases, being the most common adolescent type.^2^ ^,^ ^3^ The prevalence of AIS is 2 to 3% in individuals that range between 11 to 17 years old,^4^ and it affects mainly females.^5^

There is a correlation between the intensity of the scoliotic deformity and changes in lung function: the higher the curve, the worse the lung function.^6^ The respiratory impairment is often restrictive, being more common in cases of thoracic scoliosis. The three-dimensional deformity that affects the chest generates diaphragmatic dysfunction, compression of the lung parenchyma and progressive decrease in lung volumes and capacities.^7^ ^,^ ^8^

The Cobb method is the radiographic measurement most used to assess the magnitude of scoliosis.^9^ Currently, there are other ways to assess postural and chest wall changes objectively using photographic techniques, called digital photogrammetry.^10^ Among the new digital photogrammetry techniques available for postural and chest wall analysis, the Postural Assessment Software (PAS) stands out, a non-invasive, free and easy-to-use program, also reproducible.^11^

There is concern in finding new tools to assess the progression of scoliosis deformity using non-invasive methods in order to avoid the harmful effects of ionizing radiation. We believe that the PAS can be used as an adjunct to clinical assessment and, therefore, may prevent radiographic exposures in patients who apparently have no clinical worsening curves. Thus, we aimed to evaluate chest wall shape, using the photogrammetry method, lung function and respiratory muscle strength in patients with AIS and to compare these results with healthy subjects’ characteristics. Furthermore, we correlated chest wall shape with the Cobb method and lung function in patients with AIS.

## METHOD

The study was conducted in a cross-sectional design from March 2014 to February 2016. We enrolled AIS patients classified as Lenke type I,^12^ with the main thoracic curve to the right, including both genders aged between 11 and 18 years old. Patients from the orthopedic clinic of a local hospital underwent a radiographic evaluation to quantify the Cobb angles. The chief doctor of the spine outpatient clinic evaluated the following Cobb angles: main thoracic, proximal thoracic and lumbar. We excluded patients with history of heart, lung or neuromuscular disease, congenital malformations or other musculoskeletal disorders.

Healthy adolescents, with age, weight and height range similar to AIS patients, were recruited by advertisements in local newspapers for screening and physical therapy evaluation. A single examiner, according to the Brazilian Society of Orthopedics and Traumatology recommendations,^13^ performed the clinical screening of the control group. The Adam’s forward bend test was the primary test used to exclude subjects with any spinal deformity. In addition, we performed subjective postural assessment of the following parameters: shoulders, scapulae and pelvis alignments.

All the study participants and caregivers signed the informed assent and the consent form. The local Ethics Committee approved the present study.

We evaluated 78 participants, among which 47 had been previously diagnosed with AIS and 31 were healthy individuals represented. Among the 47 AIS patients, 17 were excluded; one due to an asthma diagnosis, while the others did not belong to Lenke type I classification (three patients with Lenke type 2, two patients with Lenke type 3, five patients with Lenke type 5, and three patients with Lenke type 6) and three patients did not have the main thoracic curve to the right. The excluded patients presented demographic and anthropometric characteristics similar to the included patients. Eleven participants were excluded from the control group due to postural abnormalities detected at the screening, such as presence of gibbosity in the Adam’s forward bend test, shoulders and scapular asymmetry and Thales triangle asymmetries.

Thus, 30 patients and 20 healthy subjects composed the final sample. We performed the post-hoc power analysis, taking in consideration the mean (SD) of the thoracic marker A2 from the groups, with the effect size of 1.53 and the alpha error of 0.05. This calculation resulted in a sample power higher than 90%.

After the individuals were screened, the evaluations followed the sequence presented: anthropometric assessment, respiratory evaluation and the chest wall evaluation (photogrammetry). The weight was measured to the nearest 0.1 kg, and height was measured to the nearest 0.5 cm. The body mass index was calculated by the body mass divided by the square of the body height (kg / m^2^).^14^

Based on the Brazilian Thoracic Association (BTA) statement,^15^ we measured the maximum inspiratory (MIP) and expiratory (MEP) pressures. The respiratory pressures were quantified by a manovacuometer (MVD 300, GlobalMed, São Paulo, SP, Brazil), and the maneuver was performed from the functional residual capacity. The pulmonary function was assessed using a spirometer (Spiropalm; Cosmed, Pavona di Albano, Italy), following the BTA recommendations.^15^ The forced expiratory volume in the first second (FEV_1_), the forced vital capacity (FVC) and the FEV_1_/FVC ratio were analysed in absolute values and percentage of predicted values.^16^

**Figure 1:**
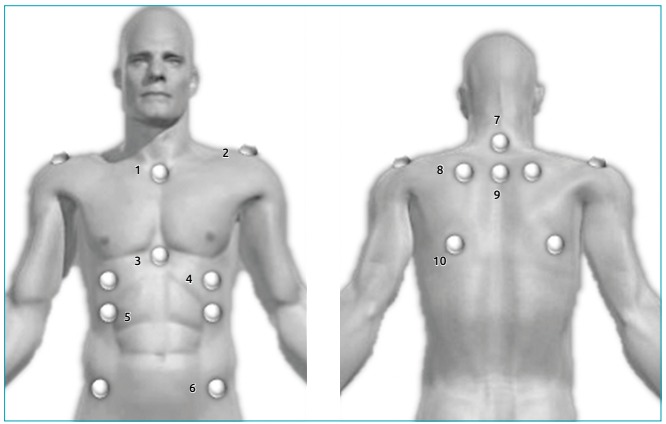
1: manubrium; 2: acromion; 3: xiphoid process; 4: point below the nipple (half the distance between nipple and last false rib); 5: last false rib (intersection of the nipple line with the last false rib line); 6: anterior superior iliac spine (ASIS); 7: spinous process of C7; 8: superior angle of the scapula; 9: spinous process of T3; 10: inferior angle of the scapula.

The chest wall deformities were evaluated by a photogrammetry method, using the postural assessment software (PAS). The PAS can be accessed for free on the website (http://demotu.org/sapo/). A professional tripod was positioned half the height of the individual, where a digital camera (Cyber-Shot DCS-W300; Sony Corporation, Tokyo, Japan) was positioned parallel to the ground. A plumb line marked 1 meter was placed on the ceiling of the room to calibrate the photos in the upright position. The pictures were recorded in the anterior and posterior view and left and right sides. The position of the feet was marked using an ethylene vinyl acetate (EVA) carpet. In order to mark the anatomical points of the chest region, a half-sphere of styrofoam balls of 25 mm diameter and double-sided adhesive tape were used. The anatomical points are shown in Figure 1.

The anatomical points used were based on the PAS protocol, except point 1^11^ and points 3, 4 and 5, that were created by our research team. The analysis of anatomical points was always performed by the same examiner and followed the steps: opening the photo, zooming 75%, calibrating the image and analysing linear and angular measurements between the previously marked anatomical points. We evaluated the thoracic markers by angles (A) and distances (D) as follows:

- A1: right acromion / manubrium / left acromion;
- A2: right acromion / xiphoid / left acromion;
- A3: last false right rib / xiphoid / last false left rib;
- A4: angle formed between the outer point of the smallest waist circumference and its upper and lower edges;
- A5: point below the nipple / inferior angle of the scapula / right and left acromion;
- A6: C7 / acromion right and left / T3;
- A7: angle formed by the intersection of the tangent segments of the upper and lower scapulae angles;
- D1: xiphoid - last false rib on the right and left sides;
- D2: manubrium - last false rib on the right and left sides;
- D3: xiphoid - anterior superior iliac spine on the left and right sides

**Figure 2:**
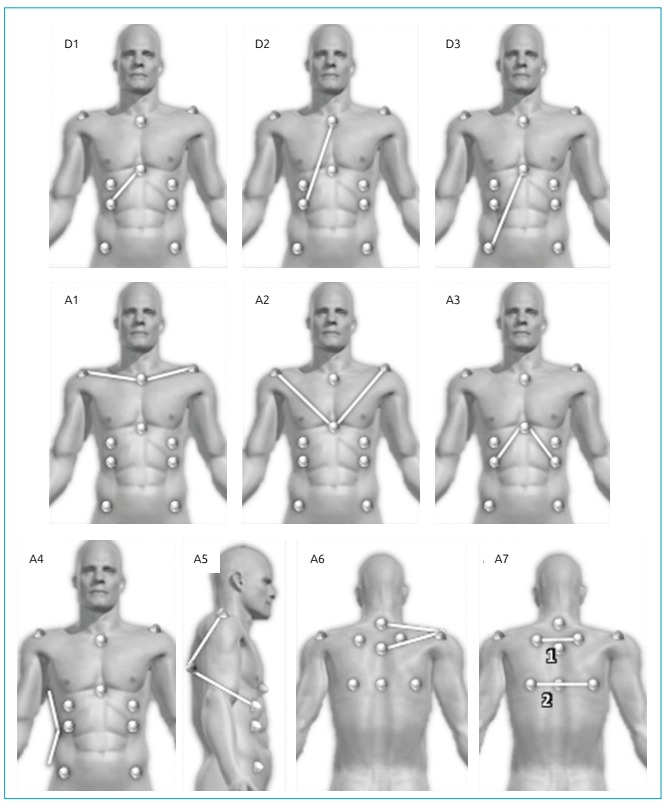
D1: xiphoid - last false rib on the right and left sides; D2: manubrium - last false rib on the right and left sides; D3: xiphoid - anterior superior iliac spine on the left and right sides; A1: right acromion / manubrium / left acromion; A2: right acromion / xiphoid / left acromion; A3: last false right rib / xiphoid / last false left rib; A4: angle between the deepest point of the waist and upper and lower edges of the waist; A5: point below the nipple / inferior angle of the scapula / right and left acromion; A6: C7 / acromion right and left / T3 ; A7: angle formed by the intersection of the tangent segments of the upper and lower scapulae angles.

All the As and Ds are illustrated in Figure 2 and were created by our team, except the A1 angle, which was reproduced from the study of Davidson et al.^11^

As the thoracic marker A4 was measured from the deepest point of the waist and its upper and lower edges, a tool called *edge detector*, available in the software, was used to evaluate this angle. This feature enhances the contrast of the picture, allowing better visualization of the edges in order to provide an accurate and reliable analysis. To evaluate the distances (D1, D2 and D3), we analysed the relationship between right and left sides (D1R/D1L, D2R/D2L and D3R/D3L).

Descriptive analysis was expressed as mean and standard deviation and frequency and percentage. The normality of variables was investigated by Kolmogorov-Smirnov test. To differentiate the groups (control and scoliosis) regarding changes in thoracic and respiratory variables, the unpaired Student T test was used, since the variables were normally distributed. To study the correlation between chest photogrammetry and pulmonary function, respiratory muscle strength and Cobb angles, the Pearson’s correlation coefficient was calculated. The probability of alpha error was set at 5% for all analyses.

## RESULTS

The demographic and anthropometric characteristics, lung function and respiratory muscle strength of the 50 subjects evaluated and the mean Cobb angles of patients with AIS analysed in this study are shown in Table 1, being the groups homogeneous in relation to gender, age, height, body mass and body mass index.

**Table 1:** Demographic, anthropometric, Cobb angle, muscular strength and lung function values assessed in the participants.

Variables	AIS (n=30)	Controls (n=20)	p-value
Female	27 (90%)	17 (85%)	0.670
Age (years)	14.3±2.1	14.6±2.4	0.730
Height (m)	1.58±0.07	1.60±0.07	0.410
Weight (kg)	47.6±6.9	50.4±9.1	0.500
BMI (kg/m^2^)	18.8±2.2	19.4±2.5	0.410
PT Cobb (º) (n=22)	24.9±11.3		
MT Cobb (º)	48.6±19.3		
L Cobb (º) (n=23)	32.6±10.8		
MIP (cmH_2_O)	63.0±23.8	77.5±24.4	0.044*
MEP (cmH_2_O)	60.6±32.4	72±24.6	0.168
FVC (L)	2.68±0.6	3.26±0.64	0.003*
FVC (% predicted)	85.3±14.2	96.8±13.3	0.006*
FEV_1_ (L)	2.34±0.55	2.81±0.62	0.011*
FEV_1_ (% predicted)	82.3±15.1	93.4±13.2	0.010*
CPF (L)	5.75±1.36	6.74±1.58	0.032*
FEV_1_ / FVC (%)	87.2±7.7	85.9±7.7	0.576

AIS: adolescent idiopathic scoliosis; n: sample size; BMI: body mass index; PT: proximal thoracic; MT: main thoracic; L: lumbar; MIP: maximal inspiratory pressure; MEP: maximal expiratory pressure; FVC: forced vital capacity; FEV_1_: forced expiratory volume in the first second; CPF: cough peak flow; *statistically significant difference (p<0.05).

The chest wall evaluation by photogrammetry showed significant increase in the thoracic markers A2, A7, and significant decrease in A4L and D1R/D1L in AIS group compared to control, as shown in Table 2.

**Table 2:** Chest wall assessment by photogrammetry in adolescent idiopathic scoliosis (AIS) and control groups.

Thoracic markers	AIS (n=30)	Controls (n=20)	p-value
Angles (degrees)			
A1	171.8±11.2	168.8±9.4	0.314
A2	88.4±8.6	78.9±6.2	< 0.001*
A3	87.9±13	90.6±9.9	0.413
A4R	161.5±6.9	158.6±7.6	0.179
A4L	144.6±9.6	158.3±6.8	<0.001*
A5R	75.2±9.1	78.2±6.7	0.182
A5L	82.9±9.8	78.3±6.1	0.051
A6R	17.4±4.4	18.4±3.5	0.414
A6L	17.4±4.0	18.6±3.5	0.257
A7	5.3±4.1	2.8±1.5	0.005*
Distances (cm)			
D1R/D1L	1.03±0.12	1.11±0.11	0.023*
D2R/D2L	1.02±0.46	1.04±0.47	0.081
D3R/D3L	1.00±0.57	1.02±0.03	0.195
Correlations		r	p-value
A2	Proximal thoracic Cobb (º)	0,47	0,028*
A2	Main thoracic Cobb (º)	0,50	0,005*
D1R/D1L	Main thoracic Cobb (º)	-0,40	0,030*

n: sample size; A1: right acromion / manubrium / left acromion; A2: right acromion / xiphoid / left acromion; A3: last false right rib / xiphoid / last false left rib; A4: angle between the deepest point of the waist and upper and lower edges of the waist; A5: point below the nipple / inferior angle of the scapula / right and left acromion; A6: C7 / acromion right and left / T3; A7: angle formed by the intersection of the tangent segments of the upper and lower scapulae angles; D1: distance between the xiphoid and last false rib; D1R/D1L: relation between D1 on the right (R) and left (L) sides; D2: distance between the manubrium and the last false rib; D2R/D2L: relation between D2 on the R and L sides; D3: distance between the xiphoid and the anterior superior iliac spine; D3R/D3L: relation between D3 on the R and L sides; cm: centimeters; p: significance level; *statistically significant difference (p<0.05); r: correlation coefficient.

There were moderate positive correlation between the thoracic marker A2 and the main and proximal thoracic Cobb angles (r=0.50, r=0.47, respectively), and moderate negative correlation between the thoracic marker D1R/D1L and the main thoracic Cobb angle (r=- 0.40), as shown in Table 2.

The MIP was found to be lower in patients with AIS when compared to healthy subjects. In the assessment of pulmonary function, all variables showed decrease in patients with AIS compared to healthy subjects, with the exception of the FEV_1_/FVC ratio, as shown in Table 1. There were moderate positive correlations between FEV_1_ and the thoracic markers D3R (r=0.47, p=0.009) and D3L (r=0.40, p=0.010).

## DISCUSSION

This study consisted on assessing chest wall shape in AIS patients using the photogrammetry method. In order to quantify the chest wall alterations, we have created some angles (A) and distances (D), named thoracic markers. We noted that this method was capable to detect changes in the thoracic markers A2, A7 and D1 in AIS patients when compared to healthy subjects. Furthermore, we found that the thoracic markers A2 and D1R/D1L correlated with Cobb angles.

The photogrammetry method used in our study was the postural assessment software (PAS). We suggested an interpretation for each one of them:

- A1, A2 and A3: tilt and rotate the ribs and sternum;
- A4: deviation and lateral inclination of the trunk;
- A5: thoracic kyphosis and/or presence of thoracic spinal deformity;
- A6: elevation of the shoulders;
- A7: uneven shoulder blades;
- D1 and D2: rotation of the chest wall;
- D3: rotation of the chest wall relative to the pelvis.

We observed increase in thoracic markers A2, A7 angles and decrease in A4L angle and the D1R/D1L relationship in individuals with AIS when compared to healthy subjects. The increase of the A2 angle in AIS group may be due to vertebral rotation, in which the vertebral body turns to the convex side and the spinous process runs to the concave side. Thus, the ribs are deformed by rotating backwards and upwards on the convex side and forward on the concave side.^6^ In our study, we also found positive correlation between the thoracic marker A2 and the main thoracic Cobb angle, showing that this marker was capable to detect rotation and inclination of the ribs.

The thoracic marker A7 was increased in AIS patients when compared to healthy individuals. According to the Brazilian Society of Orthopaedics and Traumatology, patients with AIS classified as Lenke I, with deviation of the main thoracic curve to the right, have right scapula protrusion, and the right scapula is higher than the left one in these patients; so we suggested that this marker can detect the deflection of the shoulder blades that is characteristic in these patients.

The A4L angle in individuals with AIS showed significant decrease when compared to the healthy subjects. When comparing the mean values of the right (161.5±6.9) and left (144.6±9.6) A4 angles in the AIS group, we observed that the left side angle was significantly lower (p<0.001). It shows that this population presented inclination of the trunk to the left, and that this angle was able to detect this change. This result corroborates the Brazilian Society of Orthopaedics and Traumatology assessment criteria describing the inclination of the trunk to the left as one of the individualities of patients with this classification type of scoliosis.

The D1R/D1L relationship was lower in the AIS group in comparison with the control group. As the ribs follow the vertebral rotation, rotating backwards and upwards on the convex side and turning forwards to the concave side,^6^ that may have caused the reduction in D1R and the increase in D1L, leading to a decreased relationship. We can infer that the relationship between D1R and D1L markers, due to its anatomical location, was capable to detect the rotation of the chest wall. Another result that supports this assumption is the fact that the main thoracic Cobb angle has presented negative correlation with the D1R/D1L, showing that the greater the thoracic deformity is, the smaller the D1R/D1L is.

We suggested that the decrease in A5 thoracic marker could detect the presence of spinal deformity and/or thoracic kyphosis. As the patients enrolled in this study presented right gibbosity, we expected to find the A5R decreased. Analysing this marker on both sides, we noted significant decrease (p<0.001) on the right (75.2±9.1) compared to the left (82.9±9.8) side, showing that the A5 marker was capable to detect the presence of asymmetry between the sides.

We also found positive correlation between the proximal thoracic Cobb angle and the A2 thoracic marker. Despite the average of the proximal thoracic Cobb angle was approximately 25º, we believe that this angle has been sufficient to change the axis of the cervical spine, and the A2 thoracic marker was able to represent the change. Probably, we found no correlation of angles and distances with thoracic lumbar curve due to the absence of a marker located in this region.

We identified correlation between the thoracic markers D3 at both sides and the predicted value of FEV_1_, showing that the shorter the D3 distance, the lower the FEV_1_. Accordingly, we suggested that the D3 thoracic marker detects rotation of the chest wall, and this rotational component may have caused distortion of the airways, causing increased resistance and reduced FEV_1_.

The decreased MIP, FVC and FEV_1_ in AIS individuals were correlated to decreases in muscle strength and lung volumes when compared to healthy subjects. Studies have shown that thoracic curves have worse prognosis, because the rotation of the chest wall and its effect on the respiratory system. It has been shown negative correlation between magnitude of the scoliotic curve and the lung function.^6^ ^,^ ^17^ However, some studies claim that impaired lung function are clinically relevant when patients present Cobb angles greater than 70º.^8^ ^,^ ^18^

Despite the FVC and FEV_1_ were decreased in our patients with AIS, the predicted values of FVC and FEV_1_ in this population are within the normal range, indicating no restrictive ventilatory defect. This can be explained by the fact that the average main thoracic Cobb angle in our study was 48º, which is ranked as moderate scoliosis. Thus, even with reduction in FVC and FEV_1_, these values did not generate clinically important changes.

Reduced MIP found in patients with AIS in our study corroborates with studies that assessed patients with thoracic scoliosis and showed that these patients have diaphragmatic dysfunction related to three-dimensional deformity of the chest, which leads to inspiratory muscle weakness.^8^ ^,^ ^19^

We decided to use the PAS method for its low cost, being easy to use and capable of generating databases. Several studies have shown that the reproducibility of the PAS method is reliable.^10^ ^,^ ^20^ ^,^ ^21^ ^,^ ^22^ ^,^ ^23^ ^,^ ^24^ In our study, we evaluated mainly thoracic markers, using anatomical landmarks of the PAS protocol (http://demotu.org/sapo/) and thoracic markers used by Davidson et al.^11^ In that study, the reproducibility of the created angles was excellent, with intra-class correlation coefficient (ICC) values ranging 0.83 to 0.95. Therefore, we believe that the measures are reliable and reproducible, since we followed a validated protocol.

We consider that this technique should be used in conjunction with Cobb angle and not to replace it. The gold standard test for monitoring patients with scoliosis is the Cobb method, which uses plain radiography. During the growth phase, two to three radiographic studies are required, on average, by year. Radiographs are usually panoramic and have high radiation load. It is estimated that a radiograph of the lumbar spine has up to 75 times more radiation than a chest x-ray.

Although radiography is the gold standard modality in the diagnosis of scoliosis and an important tool in monitoring the disease, there must be a concern to reduce the number of radiographs of each subject to minimize costs and exposure to radioactivity.^25^ ^,^ ^26^

Individuals with AIS undergoing radiographic examinations are young and, therefore, more sensitive to ionizing radiation. Ionizing radiation is known to cause cell death and genetic mutation, and it is associated with cancerous and noncancerous disease.^27^ In fact, excessive amount of ionizing radiation exposure in children with scoliosis has led to the development of breast cancer and increased mortality.^28^ ^,^ ^29^

Currently, there is concern about finding new ways to evaluate the changes of scoliosis, using non-invasive methods without the harmful effects of ionizing radiation. We believe that the PAS may be a useful method to screen the need to use the X-ray, since the physical examination may not have the sensitivity to detect subtle changes in curves.

The clinical evaluation of scoliosis is made by the Adams’ test and evaluation of the trunk asymmetry, both subjective criteria.^30^ ^,^ ^31^ We believe that the PAS can be used as an adjunct to clinical assessment and, therefore, may prevent radiographic exposures in patients who apparently have no clinical worsening curves.

The advantage of PAS method is that it can be used during routine assessment, in which the patient can be photographed after the anatomical landmarks are attached. Being a practical method, it allows the evaluator to measure angles and to compare them with earlier photos that can be electronically stored, becoming an objective way of assessing the deformity evolution. As an example, we can use the thoracic marker A2, which showed better correlation with the main thoracic Cobb angle and showed greater difference when compared to healthy adolescents. Moreover, in another study from our group (data in press) we observed that thoracic marker A2 showed the greatest change after scoliosis surgical treatment, proving it to be an important marker associated with deformity. Therefore, we believe that thoracic marker A2 may be a useful tool, complementing the clinical evaluation, and it may prevent at least one x-ray per year.

One limitation of this study was the fact that the PAS method promotes an evaluation of the photographs in two dimensions, and scoliosis is a three-dimensional deformity of the spine. However, we found results that suggest the presence of the rotational component of the chest wall using this method. Therefore, we consider this fact did not affect the evaluation of the rotational component of the deformity.

We concluded that the photogrammetry method using PAS was able to detect chest wall changes in AIS patients, besides presenting correlation between Cobb angles and lung function. Patients with AIS present reduced pulmonary function and inspiratory muscle strength. Thus, we suggested that the PAS can be used as an adjunct to clinical assessment, preventing radiologic exposures in patients who apparently have no clinical worsening curves.
